# Diverticulitis and Its Treatment

**Published:** 1920-03-13

**Authors:** 


### A NEW SURGICAL DISEASE.

### Diverticulitis and its Treatment.

Diverticulitis, or inflammation of a sacculus in the intestine, has been the subject of recent discussion at the Royal Society of Medicine; it is a disease which rarely occurs in early life, its prevalence reaching a maximum between the ages of sixty and seventy-five years. The pathology, diagnosis, and treatment of this condition are as yet not fully understood, and a vast amount of patient work will be required before its position as a clinical entity can be established. There is even a certain amount of dispute among pathologists as to the correct nomenclature for the condition; most authorities accept the term "diverticulitis," but there are several reasons for taking the suggestion of Professor Rutherford Morrison in calling the disease "sacculitis." Diverticulitis suggests an inflammation of a congenital sacculation such as Meckel's diverticulum, whilst, as a matter of fact, the inflammation usually occurs in or around a sacculus of acquired origin. This, however, is a question for the pathologists to decide. In the opinion of Dr. W. H. Maxwell Telling, of Leeds, who was the first surgeon in England to analyse and classify his cases, constipation is an important predisposing cause, and a co-existing flatulent distension, plays a part that cannot be overlooked, and needs more study.

A knowledge of the anatomy of the various intestinal diverticula is of the first importance in understanding the secondary pathological processes, and also in detecting them at the time of operation.

The pelvic colon is the place where they are chiefly found; and we may mention, in passing, that unless carefully looked for they are usually missed entirely. They are small, varying from minuteness up to the size of one's little finger: their apertures are minute and usually occur in two rows, mostly opposite the appendices epiploicae and often completely concealed by the rugose mucous membrane. Once a sacculus has formed, several possible consequences are described, viz.—(1) It tends to enlarge; (2) it tends to harbour fæcal or other harmful contents; and (3) as a consequence, it tends to undergo various secondary pathologic processes. The main secondary processes which may occur are: (1) Ulceration of the mucosa of the diverticulum. (2) Perforation as a consequence. (3) Adhesion to other structures which, when combined with perforation, leads to fistulous communication. (4) Peridiverticular fibrous hyperplasia, leading to tumour formation; and, as a sequential result, stenosis of the bowel.

### Symptoms and Diagnosis.

The symptoms of diverticulitis cannot be described as classical, for in the main they imitate those of appendicitis, though the symptoms in this case are generally localised in the left lower abdominal quadrant. Thus there may be a sudden general peritonitis, determined probably by a perforation; or, on the other hand, all the appendicitis-like symptoms of inflammation, ranging from a mere plastic peritonitis to abcess formation, either localised or diffuse. In 1906 Sir Berkeley Moynihan remarked upon the mimicry of carcinoma occasioned by chronic inflammations of the colon, especially that due to tubercular disease. The same may be said about diverticulitis; both this condition and carcinoma cause a stenosis of the bowel, and the resulting obstructive symptoms are exceedingly difficult to distinguish. Cases, often recorded in the past, where a colostomy has been performed for a supposed inoperable carcinoma, and in which the patient has survived for many years with gradual diminution of the tumour, were in all probability instances of chronic diverticulitis.

A good deal of clinical experience is necessary to make the diagnosis of 'diverticulitis" with anything like the certainty with which other abdominal inflammations are recognised, although Professor Erdmann has gone so far as to say that the occurrence of left-sided pain with mass, but with tenderness and rigidity, is practically pathognomonic of diverticulitis. This is the extreme view, favoured by the main supporters of the almost exclusive rôle of diverticula in inflammatory troubles in the left abdomen. Professor Fitz in 1912 said that the cases comprised in this "inflammatory group" made what must be recognised as a new disease of the lower abdomen—diverticulitis of the left quadrant, analogous to appendicitis of the right quadrant.

### A Cause of Vesico-Colic Fistula.

In a recent review by Bryan in the *Annals of Surgery*, the statement is made that "diverticulitis is the most frequent cause of that distressing condition—vesico-colic fistula." Sir Berkeley Moynihan also emphasises this fact in a paper in which he says: "In cases where a hard growth in the intestine is accompanied by the passage of flatus and fæces by the urethra, a diagnosis of carcinoma seems irresistible; yet the probability is that the 'growth' would be simple, and that the cause of the fistula would be a false diverticulum, which had burrowed its way through all the coats of the bowel, and thence through the wall of the bladder, which had become adherent."

Experience has abundantly confirmed the truth of this statement, and its recognition has been followed by some brilliantly successful cases at Leeds.

### Treatment.

This is comprised in a single word—Surgery—unless operative interference is specially contraindicated by the general condition of the patient or a serious concurrent disease.

At the operation all diverticulum-bearing gut should be removed by resection and an intestinal

**A New Surgical Disease**—(*continued*).

anastomosis performed. Extreme care should be taken in handling the intestines, as at least one instance has occurred of rupture of a diverticulum by surgical trauma. As a post-operative complication there seems to be a special liability to peritonitis, possibly due to the chronic infection of the inflamed tissues in some cases.

CONCLUSIONS.

The good results already obtained in certain bladder fistulæ should stimulate surgeons to deal with such cases. No case of supposed cancer of the lower bowel must in future be regarded as inoperable, either before or actually at the time of laparotomy, unless diverticulitis has been remembered, fully considered, and systematically investigated. As prevention is better than cure, it should be the duty of the family medical attendant to encourage men and women, after middle life, to have efficient sets of teeth, and warn them against gobbling indigestible food, or bolting hot and unpalatable morsels; advice which would be good, whether the new theory be correct or not.

